# First report of molecular detection of fluoroquinolone resistance-associated *gyrA *mutations in multidrug-resistant clinical *Mycobacterium tuberculosis *isolates in Kuwait

**DOI:** 10.1186/1756-0500-4-123

**Published:** 2011-04-14

**Authors:** Noura M Al-Mutairi, Suhail Ahmad, Eiman Mokaddas

**Affiliations:** 1 Department of Microbiology, Faculty of Medicine, Kuwait University, Kuwait

**Keywords:** *M. tuberculosis*, Fluoroquinolone resistance, *gyrA *mutations, Kuwait

## Abstract

**Background:**

Nearly 5% of all *Mycobacterium tuberculosis *strains worldwide are resistant at least to rifampicin and isoniazid (multidrug-resistant tuberculosis, MDR-TB). Inclusion of a fluoroquinolone and an injectable agent (kanamycin, amikacin or capreomycin) in multidrug therapy is crucial for proper treatment of MDR-TB. The incidence of MDR-TB in Kuwait is ~1%. MDR-TB strains additionally resistant to fluoroquinolones and injectable agents are defined as extensively drug-resistant (XDR-TB) strains and have been detected in >55 countries. Infections with XDR-TB strains have very poor prognosis. This study detected the occurrence of *gyrA *mutations associated with fluoroquinolone resistance among MDR-TB strains in Kuwait.

**Findings:**

Direct DNA sequencing of quinolone resistance-determining region of *gyrA *gene was performed to detect fluoroquinolone resistance-associated mutations in 85 MDR-TB strains isolated from 55 TB patients and 25 pansusceptible *M. tuberculosis *strains. For isolates exhibiting *gyrA *mutations, 3'-end of *rrs *(16S rRNA) was sequenced for the detection of XDR-TB. Fingerprinting of fluoroquinolone resistant MDR-TB strains was performed by detecting mutations in three (81 bp hot-spot, N-terminal and cluster II) regions of *rpoB*, *katG *codon 315 and *inhA*-regulatory region, polymorphisms at *gyrA *codon 95 and *katG *codon 463 by DNA sequencing and by double-repetitive-element PCR for determining strain relatedness. None of the pansusceptible but six of 85 MDR-TB strains contained *gyrA *mutations. Only *gyrA *codon 94 was mutated in all six (D94A in one and D94G in five) strains. Three of six mutant strains were recovered from the same patient while three other strains represented individual patient isolates. Fingerprinting studies identified all individual patient isolates as epidemiologically distinct strains. All six strains with a *gyrA *mutation contained wild-type *rrs *sequence.

**Conclusions:**

Although fluoroquinolones are generally not used for chemotherapy of TB and drug susceptibility testing for second-line drugs is not carried out in Kuwait, four of 55 (7%) individual patient MDR-TB strains contained mutations in *gyrA *gene. The data advocate routine drug susceptibility testing for this important second-line drug for proper management of MDR-TB in Kuwait. Lack of mutations in 3'-end of *rrs *gene that confer resistance to injectable agents reduce the likelihood of occurrence of XDR-TB, at present, in Kuwait.

## Background

Tuberculosis (TB), causing nearly 9 million active disease cases and two million deaths worldwide every year, is a major public health issue [1]. Increasing resistance of *Mycobacterium tuberculosis *strains to most-effective (first-line) anti-TB drugs and strong association of human immunodeficiency virus (HIV) pandemic with active TB disease are the two major contributors to the current global burden of TB [1-3]. Incomplete/improper treatment of TB patients leads to evolution of drug-resistant *M. tuberculosis *strains due to chromosomal mutations in genes encoding drug targets [4]. Sequential accumulation of mutations in target genes generate multidrug-resistant (resistant at least to rifampicin and isoniazid) *M. tuberculosis *(MDR-TB) and extensively drug-resistant (additionally resistant to fluoroquinolones and an injectable anti-TB agent such as kanamycin, amikacin or capreomycin) *M. tuberculosis *(XDR-TB) strains [4,5]. While proper treatment of drug-susceptible TB has ≥95% cure rate, effective treatment of MDR-TB is difficult in developing countries as it is heavily dependent on rapid diagnosis, supervised aggressive therapy with several (5-6) expensive, toxic and less efficacious (second-line) drugs for 18-24 months [4-7]. Inclusion of a fluoroquinolone and an injectable agent in multidrug treatment regimens have a more favorable outcome in the treatment of MDR-TB [4-8]. Treatment of XDR-TB is far more difficult even in developed countries while in developing countries, XDR-TB is virtually an untreatable disease [8-10]. The prognosis of XDR-TB in HIV-coinfected TB patients is extremely poor, with fatality rates varying from ~30% in developed countries to nearly 100% in developing countries [8-11]. Hence, all efforts should be made to successfully cure the existing MDR-TB cases to avoid the emergence of XDR-TB [4,6,8,12].

Fluoroquinolones (FQs), particularly new generation compounds (such as moxifloxacin and gatifloxacin) and injectable aminoglycosides (kanamycin and amikacin) and cyclic peptide (capreomycin) have excellent bactericidal activity against *M. tuberculosis *and are crucial for proper management of MDR-TB patients [4,8,12,13]. Widespread emergence of MDR-TB has accelerated development of rapid drug susceptibility testing (DST) procedures for these important second-line drugs to help design effective treatment strategies. The cellular target of FQs in *M. tuberculosis *is DNA gyrase, a type II topoisomerase consisting of two A and two B subunits encoded by *gyrA *and *gyrB *genes, respectively [14]. Mutations in a small region of *gyrA*, called quinolone resistance-determining region (QRDR) and, less frequently, in *gyrB *are the primary mechanism of FQ resistance in *M. tuberculosis *[14-16]. Analysis of QRDR alone by genotypic tests has been suggested as sufficient for rapid identification of vast majority of FQ-resistant *M. tuberculosis *strains as additional targeting of *gyrB *did not enhance the sensitivity significantly [16,17]. The molecular basis of resistance of *M. tuberculosis *strains to injectable agents such as aminoglycosides (kanamycin and amikacin) and cyclic peptide (capreomycin) has also been determined [4,18,19]. Nearly 90% to 95% of *M. tuberculosis *strains resistant to one or more of the injectable agents contain mutations near the 3'-end of *rrs *(16S rRNA) gene involving nucleotide positions A1401, C1402, and G1484 [4,17-19].

The rate of MDR-TB is quite low (~1%) and FQs and injectable agents are rarely used for chemotherapy of TB in Kuwait [20]. This study was carried out to detect the occurrence of *gyrA *mutations associated with fluoroquinolone resistance among MDR-TB strains. The 3'-end of *rrs *gene was also sequenced among isolates containing *gyrA *mutations to detect the occurrence of XDR-TB in Kuwait.

## Methods

### Bacterial isolates and susceptibility testing

A total of 4926 *M. tuberculosis *strains were isolated from TB patients during the study period (January 2001 to June 2008) at National Tuberculosis Reference Laboratory in Kuwait. A total of 110 *M. tuberculosis *isolates were analyzed in this study. These included 85 MDR-TB strains isolated from 55 TB patients (representing all available MDR *M. tuberculosis *strains). Twenty-five drug-susceptible *M. tuberculosis *strains (isolated from 25 patients) were also included to ensure that fluoroquinolone resistance-conferring mutations in the *gyrA *gene are not found in pansusceptible *M. tuberculosis *strains. *M. tuberculosis *H_37_Rv was used as a control. Isolation and identification of *M. tuberculosis *isolates was performed by using MGIT 960 system (Becton Dickinson) and multiplex PCR as described previously [20,21]. The phenotypic DST against first-line drugs (isoniazid, rifampicin, ethambutol, and streptomycin) was carried out by BACTEC 460 TB system as described in detail previously [20].

### DNA extraction for molecular assays

One ml of MGIT 960 culture of reference or clinical *M. tuberculosis *isolate was heated with 40 mg Chelex-100 (Sigma-Aldrich) at 95°C for 20 min and then centrifuged at 12,000 × g for 15 min [22]. For a PCR, 2 μl of supernatant was used as a source of DNA.

### Detection of mutations in QRDR of the *gyrA *gene by DNA sequencing

The QRDR of the *gyrA *gene was amplified by touchdown PCR by using GYRAF (5'- CGCAGCTACATCGACTATGCGATG-3') and GYRAR (5'-GGGATGAAATCGATGTCTCCTCG-3') as amplification primers and the reaction and thermal cycling conditions as described in detail previously [23]. The 400 bp amplicons were purified by using PCR product purification kit (Qiagen) and sequenced by using cycle DNA sequencing kit (DTCS CEQ2000, Beckman-Coulter) and GYRAFS (5'-CGGGTGCTCTATGCAATGTTC-3') or GYRARS (5'- GGCTTCGGTGTACCTCATCGCC-3') as internal sequencing primer. Although PCR products were purified, internal primers were used for sequencing of PCR amplicons to avoid interference from trace amounts of primer dimers, if still present. The detailed methodology of DNA sequencing was same as described in detail previously [23]. Nucleotide and amino acid sequences of the amplified products were compared with corresponding sequences from susceptible strain *M. tuberculosis *H_37_Rv using BLAST. DNA sequencing of QRDR also revealed the polymorphism (S95 or T95) that occurs at *gyrA *codon 95 among *M. tuberculosis *strains [24].

### Fingerprinting of MDR-TB strains carrying *gyrA *mutation

Fingerprinting of MDR-TB strains carrying *gyrA *mutation was carried out by direct DNA sequencing of hot-spot, N-terminal and cluster II regions of *rpoB *gene, *inhA*-regulatory region and *katG *codon 315 to detect mutations conferring resistance to rifampicin and isoniazid, as described in detail elsewhere [25]. The genetic group [24] of the isolates was determined by detecting the polymorphism at *gyrA *codon 95 (described above) and at *katG *codon 463 (L463 + T95, Group I; R463 + T95, Group II and R463 + S95, Group III). The presence of R463/L463 at *katG *codon 463 was detected by PCR amplification with KATG463F and KATG463R primers followed by restriction digestion with *Nci *I, to generate RFLP patterns, as described previously [26]. Further fingerprinting of the isolates was carried out by double-repetitive-element (DRE)-PCR and isolates yielding unique patters were classified as genotypically distinct strains [27].

### Detection of mutations at 3'-end of *rrs *gene by DNA sequencing

The 3'-end of *rrs *(16S rRNA) gene that is mutated in nearly 90% to 95% of *M. tuberculosis *strains exhibiting resistance to injectable agents (kanamycin, amikacin and capreomycin) [17-19] was also amplified by touchdown PCR by using 16S3F (5'-GCGATGCCGCGAGGTTAAGCGAA-3') and 16S3R (5'-CCAACAGTGTGTTGGTGGCCAA-3') as amplification primers and the reaction and thermal cycling conditions described previously [23]. The 403 bp amplicons were purified and sequenced by using 16S3FS (5'-ATCCTTAAAAGCCGGTCTCAGT-3') or 16S3RS (5'- CTCCTTAGAAAGGAGGTGATCCA-3') as internal sequencing primer. Again, internal primers were used for sequencing to avoid interference from trace amounts of primer dimers, if still present along with of PCR amplicons of *rrs *gene. The detailed DNA sequencing protocol has been described in detail previously [23]. Nucleotide and amino acid sequences of the amplified products were again compared with corresponding sequences from susceptible strain *M. tuberculosis *H_37_Rv using BLAST.

### Nucleotide sequence accession numbers

The DNA sequencing data reported in this study have been deposited in EMBL under accession numbers FR734170-FR734173.

## Results

Phenotypic DST data for 85 MDR-TB strains showed that 21 (25%) isolates were resistant to isoniazid and rifampicin only while seven (8%), 23 (27%) and 34 (40%) isolates were additionally resistant to streptomycin, ethambutol and to streptomycin and ethambutol, respectively (Table 1). Of the 55 patients yielding MDR-TB strains, only two were Kuwaiti nationals while the remaining 53 patients were expatriate workers or their family members. The countries of origin for the 53 expatriate patients included India (n = 24), Philippines (n = 10), Egypt (n = 7), Bangladesh (n = 2), Indonesia (n = 2), Syria (n = 2), Iraq (n = 2), Ethiopia (n = 1), Nepal (n = 1), Nigeria (n = 1) and Pakistan (n = 1). The date of arrival in Kuwait was not available for the expatriate patients. All *M. tuberculosis *isolates were recovered from HIV-seronegative adult TB patients between the ages of 21 to 65 years and were resistant to the indicated drugs on first isolation; however, information on prior treatment of expatriate patients with anti-TB drugs was not available. The 25 *M. tuberculosis *isolates were susceptible to all first-line drugs (pansusceptible strains).

**Table 1:** Resistance patterns and presence of fluoroquinolone resistance-associated *gyrA *mutations in 110 *M. tuberculosis *strains tested.

Resistance pattern of	No. of isolates	No. of isolates with
***M. tuberculosis *isolate**^**a**^	tested	*gyrA *mutation
None	25	0
H, R	21	0
H, R, S	7	0
H, R, E	23	4*
H, R, S, E	34	2
Total	110	6

^a^H, isoniazid; R, rifampicin, S, streptomycin; E, ethambutol

*3 of 4 *M. tuberculosis *isolates were recovered from the same TB patient

**Figure 1:**
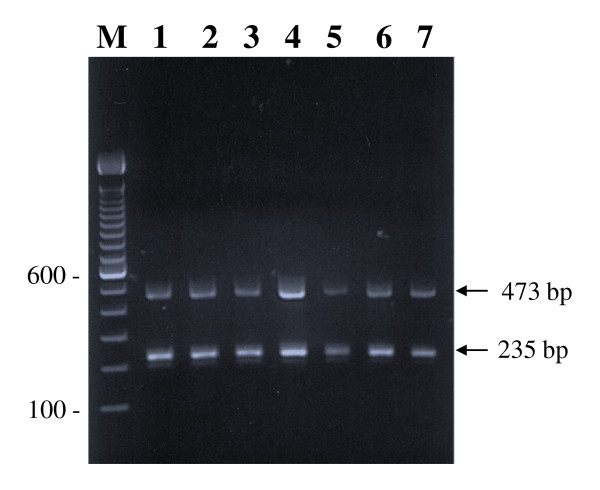
**Species-specific identification of *M. tuberculosis *isolates by multiplex PCR**. Representative agarose gel of amplicons of multiplex PCR from 7 selected multidrug-resistant *M. tuberculosis *isolates (lanes 1-7) showing *M. tuberculosis*-specific amplification of two fragments of 473 bp and 235 bp (marked by arrows) from *oxyR *and *rpoB *genes, respectively. Lane M is 100 bp DNA ladder and the position of migration of 100 and 600 bp fragments are marked.

All 110 isolates were identified as *M. tuberculosis *by a multiplex PCR that yielded 2 DNA fragments of ~473 bp and ~235 bp derived from *oxyR *and *rpoB *genes, respectively, as expected (data from seven selected MDR-TB strains are shown in Figure 1). The PCR amplification of QRDR of *gyrA *gene from reference strain *M. tuberculosis *H_37_Rv as well as from all 25 pansusceptible and 85 MDR *M. tuberculosis *strains yielded an expected amplicon of ~400 bp. The DNA sequencing data of all 25 pansusceptible *M. tuberculosis *strains showed complete concordance with wild-type *gyrA *sequence from reference strain except for polymorphism (S95 or T95) at *gyrA *codon 95, which is not associated with fluoroquinolone resistance [24]. The DNA sequencing data from 85 MDR-TB strains showed nucleotide (and amino acid) changes in QRDR of the *gyrA *gene in six isolates while the remaining 79 strains contained wild-type sequences except for *gyrA *codon 95 (Table 1). Among the six MDR-TB strains, nucleotide changes were detected only at *gyrA *codon 94, with five strains containing GAC94GGC (D94G) mutation and one isolate containing GAC94GCC (D94A) mutation. Interestingly, FQ resistance in MDR-TB strains was associated with additional resistance to ethambutol. Thus, all MDR-TB strains with gyrA mutations were resistant to three or all four first-line drugs (Table 1). Three of six FQ-resistant strains were isolated from the same patient within a period of two months while the remaining three isolates were recovered from three separate TB patients. Thus, four of 55 (7%) individual patient MDR-TB strains contained mutations in QRDR of the *gyrA *gene.

The year of isolation and results of fingerprinting studies for the four individual patient MDR-TB strains containing FQ resistance-associated *gyrA *mutations are presented in Table 2. The isolates were recovered over a four-year period (2004 to 2008) from male expatriate patients originating from India (n = 3) and Bangladesh (n = 1). Three patients developed pulmonary TB while the fourth patient had extrapulmonary form of the disease. Fingerprinting of the isolates based on specific mutations in hot-spot, N-terminal and cluster II regions of *rpoB, katG *codon 315 and *inhA*-regulatory region and genetic group analyses showed that all four isolates were distinct strains (Table 2). All four isolates also yielded unique DRE-PCR patterns. The two repeat isolates recovered from one patient (no. 3) exhibited the same fingerprinting patterns as the first isolate from this patient (data not shown).

**Table 2:** Patient's demographic data and fingerprinting data for the four individual patient multidrug-resistant *M. tuberculosis *strains containing a mutation in quinolone resistance-determining region (QRDR) of *gyrA *gene.

Patient	Nationality	Clinical	Year of	Resistance	Mutation in QRDR	**Fingerprinting data from analysis of**^**b**^		
**no**.		specimen	isolation	**pattern**^**a**^	of *gyrA *gene	***rpoB***^**c**^	***inhA*-RR**^**d**^	**Genetic group**^**e**^
1	Indian	Pleural fluid	2004	H, R, S, E	GAC94GGC/D94G	Wild-type^f^	WT	I
2	Bangladeshi	Sputum	2006	H, R, S, E	GAC94GCC/D94A	H526Q + D516G	WT	III
3*	Indian	Sputum	2006	H, R, E	GAC94GGC/D94G	I572F	WT	I
4	Indian	Sputum	2008	H, R, E	GAC94GGC/D94G	S531L	-17G→T	I

^a^H, isoniazid; R, rifampicin, S, streptomycin; E, ethambutol

^b^All four were male patients and all individual patient isolates contained AGC315ACC mutation in *katG *codon 315 and yielded unique patterns in DRE-PCR

^c^Three (hot-spot, N-terminal and cluster II) regions of *rpoB *gene were sequenced to detect rifampicin resistance-conferring mutations and codon numbering system of *rpoB *gene from *Escherichia coli *is used [4]

^d^*InhA*-RR, *inhA *regulatory region sequencing data

^e^Genetic groups were assigned based on polymorphisms at *katG *codon 463 and *gyrA *codon 95(L463/T95, Group I; R463/S95, Group III)

^f^This isolate either contained a mutation in other regions of the *rpoB *gene or in other genes involved in rifampicin resistance [25]

*Two repeat isolates recovered from this patient exhibited identical patterns

## Discussion

Kuwait, an Arabian Gulf country with nearly 25 cases per 100, 000 population is a low TB incidence country [28]. However, it has a large expatriate population originating from TB endemic countries of South-Southeast Asia. Nearly 600 patients are diagnosed with mycobacterial infections every year. Nearly 95% of mycobacterial infections are caused by *M. tuberculosis *(TB) while the remaining 5% are caused by non-tuberculous mycobacteria [29]. The non-tuberculous mycobacterial infections are more common among Kuwaiti patients, however, ~80% of TB patients are expatriate workers or their family members [20,29]. This is despite the fact that all expatriates are screened for TB and HIV on entry and are allowed to stay in Kuwait only if they exhibit no obvious signs (suspected lesions on chest radiograph) of active TB disease or previous exposure to active disease. Furthermore, all non-infectious TB cases are sent back (after anti-TB therapy of 4-8 weeks, if required) to their respective countries for further management [20]. While global proportion of MDR-TB has been estimated to be ~5.3% among all cases [2,3], only ~1% of *M. tuberculosis *isolates in Kuwait are MDR-TB strains and nearly all of these strains are isolated from expatriate patients [20]. The incidence of extrapulmonary TB in Kuwait is relatively high, accounting for 44% of all TB cases and majority of pulmonary TB patients have cavitary lesions in the upper lobes [20,28]. These findings together with the low incidence of TB in Kuwait are consistent with observations that majority of active TB disease cases in foreign-born persons occur as a result of reactivation of prior (latent) infection, usually within the first few years of their migration [30]. Previous fingerprinting studies have also shown that vast majority of MDR-TB cases among expatriate patients in Kuwait have unique patterns [31,32]. Since fingerprinting patterns are highly variable in countries that have a low incidence of active TB disease and immigrants originating from high incidence countries [33], these observations also support reactivation of previously acquired infection as the major mechanism for active TB disease in most patients in Kuwait.

The FQs play an important role in the treatment of MDR-TB since their inclusion in therapy regimens improves treatment outcome. Resistance of MDR-TB strains to FQs is associated with poor treatment outcome and is also one of two key defining conditions of XDR-TB [4,7-10,12]. Thus, there is a pressing need for rapid DST of MDR-TB strains against FQs to improve clinical management. However, like many other countries [14], routine DST for second-line drugs has not been instituted in the National Tuberculosis Control Program in Kuwait, mainly due to low rate of MDR-TB [20]. Before planning for routine phenotypic and/or genotypic DST for FQs and other second-line drugs in Kuwait, this study was carried out to detect the occurrence of *gyrA *mutations associated with FQ resistance among MDR-TB strains.

Our data showed that four of 55 (7%) individual patient MDR-TB isolates in Kuwait contained mutations in QRDR of the *gyrA *gene. Interestingly, all MDR-TB strains with a *gyrA *mutation were additionally resistant to ethambutol with/without additional resistance to streptomycin. Previous studies have also noted an association of FQ resistance in *M. tuberculosis *strains with additional resistance to several first-line drugs [15,17]. All four individual patient isolates contained a mutation at *gyrA *codon 94 which was also the most frequently mutated codon among FQ-resistant *M. tuberculosis *strains in several previous studies [15,17,34-37]. Three of four individual patient isolates with *gyrA *mutation contained D94G mutation while the fourth isolate contained D94A mutation. Studies from Germany, Taiwan and Russia have also reported that D94G is the most common mutation observed in FQ-resistant *M. tuberculosis *strains [15,17,35] while FQ-resistant strains from Japan contained D94A as the most frequent mutation [37]. Although phenotypic drug susceptibility testing for FQs was not performed in this study, the level of resistance in FQ-resistant *M. tuberculosis *strains could be inferred based on the nature and kind of *gyrA *mutations detected. Previous studies have shown that *M. tuberculosis *strains containing D94G mutation in the *gyrA *gene exhibit minimum inhibitory concentration (MIC) of ≥4 μg/ml while those with D94A mutation have MIC of 2 μg/ml for levofloxacin [37,38]. Based on these observations, it may be inferred that three of four FQ-resistant *M. tuberculosis *strains from Kuwait were highly resistant to fluoroquinolones.

Three of four patients infected with *M. tuberculosis *strains containing *gyrA *mutation originated from India while the fourth patient was from Bangladesh. Since FQs are frequently used for other bacterial infections, it is probable that many expatriate patients were exposed to these agents in their respective native countries. Recent data have shown that 24% and 9% of MDR-TB strains from India and Bangladesh, respectively, are resistant to FQs [5]. The MDR-TB strains containing *gyrA *mutations were cultured at the time of clinical diagnosis before anti-TB treatment was initiated (primary resistance) and not during treatment during a five-year period and were genotypically distinct strains. These observations together with the low incidence of active TB disease and low rates of active transmission of infection in Kuwait [20,28-30] imply that the four expatriate patients did not acquire the infection recently but were most likely latently infected with FQ-resistant strains. A limitation of the present study is that phenotypic DST for FQs was not performed. Previous studies from several geographical locations have shown that nearly 70% to 100% of FQ-resistant *M. tuberculosis *strains contain mutations in QRDR of the *gyrA *gene while FQ resistance in other isolates is associated with mutations outside of QRDR of *gyrA *gene or due to *gyrB *gene mutations [15,17,34-38]. It is, therefore, probable that phenotypic DST may have identified few (one or two) additional FQ-resistant MDR-TB strains in Kuwait. Thus, phenotypic DST should be performed as an important preliminary tool to determine the real prevalence of FQ resistance in Kuwait.

The absence of mutations in 3'-end of *rrs *gene, which confer resistance to kanamycin, amikacin or capreomycin [17-19], among the six FQ-resistant MDR-TB strains is reassuring as it decreases the possibility of the presence of XDR-TB in Kuwait. This is most likely due to very infrequent use of these injectable agents for the treatment of TB in Kuwait.

## Conclusions

The detection of fluoroquinolone resistance-associated mutations in *gyrA *gene in four of 55 (7%) individual patient MDR-TB strains strongly suggest the need for routine drug susceptibility testing for this important second-line drug for proper management of MDR-TB in Kuwait. However, lack of mutations in 3'-end of *rrs *gene that confer resistance to injectable agents is encouraging and rules out, at least for now, the presence of XDR-TB in Kuwait.

## Competing interests

The authors declare that they have no competing interests.

## Authors' contributions

SA and EM designed the study and NMA carried out the experiments. All authors contributed in manuscript writing. All of the authors have read and approved the final manuscript.
